# Accumulation in nutrient acquisition strategies of arbuscular mycorrhizal fungi and plant roots in poor and heterogeneous soils of karst shrub ecosystems

**DOI:** 10.1186/s12870-022-03514-y

**Published:** 2022-04-11

**Authors:** Yueming Liang, Fujing Pan, Zhongcheng Jiang, Qiang Li, Junbing Pu, Kunping Liu

**Affiliations:** 1 Key Laboratory of Karst Dynamics, Ministry of Natural and Resources & Guangxi Zhuangzu Autonomy Region, Institute of Karst Geology, Chinese Academy of Geological Sciences, No.50 Qixing Road, Qixing District, Guilin, 541004 Guangxi China; 2 Guangxi Key Laboratory of Theory and Technology for Environmental Pollution Control, College of Environmental Science and Engineering, Guilin University of Technology, No.12 Jiangan Road, Qixing District, Guilin, 541004 Guangxi China; 3 International Research Center On Karst Under the Auspices of UNESCO, No.50 Qixing Road, Qixing District, Guilin, 541004 Guangxi China; 4 Huanjiang Observation and Research Station for Karst Eco-Systems, Institute of Subtropical Agriculture, Chinese Academy of Sciences, Huanjiang, 547100 China

**Keywords:** Karst, Shrubland ecosystem, Soil nutrient heterogeneity, Nutrient acquisition strategies, Root trait, Arbuscular mycorrhizal (AM) fungi

## Abstract

**Background:**

Arbuscular mycorrhizal (AM) fungi and roots play important roles in plant nutrient acquisition, especially in nutrient poor and heterogeneous soils. However, whether an accumulation strategy of AM fungi and root exists in such soils of karst shrubland ecosystems remains unclear. Root traits related to nutrient acquisition (root biomass, AM colonisation, root acid phosphatase activity and N_2_ fixation) were measured in two N_2_-fixing plants (i.e. *Albizia odoratissima* (Linn. f.) Benth. and *Cajanus cajan* (Linn.) Millsp.) that were grown in heterogeneous or homogeneous nutrient (ammonium) soil with and without AM fungi inoculation.

**Results:**

Both of these plants had higher AM colonisation, root biomass and relative growth rate (RGR), but lower N_2_ fixation and root acid phosphatase activity in the rhizosphere in the heterogeneous soil environment, than that in the homogeneous soil environment. Plants grown in the AM fungi-inoculated heterogeneous soil environment had increased root biomass and root acid phosphatase activity compared with those grown in soil without inoculation. AM colonisation was negatively correlated with the N_2_ fixation rate of *A. odoratissima*, while it was not significantly correlated with the root phosphatase activity.

**Conclusions:**

Our results indicated that enhanced AM symbiosis and root biomass increased the absorptive surfaces for nutrient acquisition, highlighting the accumulation strategies of AM and root traits for plant nutrient acquisition in nutrient poor and heterogeneous soils of the karst shrubland ecosystem.

**Supplementary Information:**

The online version contains supplementary material available at 10.1186/s12870-022-03514-y.

## Background

Spatial heterogeneity of soil nutrient availability is common in terrestrial ecosystems [1]. Plants have explored different root foraging strategies to adapt to such heterogeneity [2, 3]. Arbuscular mycorrhizal (AM) fungi [4, 5] and root traits (e.g. root biomass and shoot: root ratio) [6, 7] are both needed for plant nutrient acquisition, and their variation may reflect key nutrient acquisition behaviours in the belowground environment [8–10]. For example, plants will modify their biomass allocation in heterogeneous soil environments [11], and allocate more carbohydrates belowground for root proliferation and produce more roots in high nutrient availability patches [12, 13]. Additionally, a previous study indicated that plant roots enhance their symbiosis with AM in heterogeneous soil environments, and have higher levels of AM colonisation in heterogeneous soils than in homogeneous soils [14]. These results are likely due to the plants’ improving their absorptive surfaces mainly depending on roots and AM colonisation for the acquisition of nutrients in heterogeneous soil environments. Therefore, an accumulation of nutrient foraging strategies is expected to occur between root and AM fungi in soils with heterogeneous nutrient availability.

AM fungi and root represent two important strategies for construction belowground absorptive surface area, thus examining how root and AM fungi simultaneously respond to heterogeneous in soil nutrient is important. Liu et al. [10] simultaneously tested and compared of AM colonisation and root biomass in treatments with nitrogen and phosphate addition, showed that root growth increased under nutrient additions [10]. In contrast, AM colonisation decreased, and phosphorus addition was more effective in reducing AM colonisation than nitrogen addition [10]. Previous studies have evaluated the responses of roots or AM colonisation level to the spatial heterogeneity of soil nutrient [5, 10, 15]; however, few studies have simultaneously examined how AM and roots respond to the spatial heterogeneity of soil nutrient, particularly in karst shrubland ecosystems. These ecosystems are characterised by critical factors such as: (i) a higher spatial heterogeneity of soil nutrient availability due to higher rock exposure comparing with other non-karst regions [16], and (ii) higher diversity of soil AM fungi [17]. Therefore, a clarification of the changes in both the root traits (i.e. root biomass) and AM fungi can improve our understanding the responses of plants to spatial heterogeneity of soil nutrient supply, and also provide valuable insights into belowground resource acquisition strategies in karst shrubland ecosystems.

Finally, the responses of AM fungi and roots to spatial heterogeneity in soil nutrient were closely related to other root traits, e.g. root symbiotic rhizobia and root acid phosphatase. Plant root symbiosis with rhizobia enables plants fixation nitrogen for their growth [18]. Simultaneously, phosphatase is secreted by plant roots and AM fungi, which mineralise more organic phosphorus from ester-bound forms to the orthophosphate form and thus increase plant phosphorus uptake [19–21]. Phosphorus and nitrogen are essential nutrients for plant growth, and are generally limited in karst shrubland ecosystems [22]. Thus, more attention should be paid to clarify how root symbiotic rhizobia and root acid phosphatase traits differ in their responses to spatial heterogeneity in soil nutrient availability.

In the present study, we hypothesised that: (1) N_2_-fixing plants grown in heterogeneous soils would predominantly exploit nutrients for plant growth via the allocation of more biomass to the roots and an enhanced symbiosis with AM fungi. These accumulation strategies for nutrient acquisition would thus be advantageous for plant growth in the heterogeneous and poor nutrient soils of karst shrubland ecosystems. (2) Root biomass would be higher in heterogeneous soils inoculated with AM compared with homogeneous soils, which would increase the root absorption area and thus alleviate the stress of spatial heterogeneity in soil nutrient availability on plant growth in karst shrubland ecosystems. Therefore, we conducted a potted experiment in karst shrub ecosystems of Southwest China to examine the integrated strategies for N_2_-fixing plant species (*Albizia odoratissima* and *Cajanus cajan*), that are widely distributed in karst shrubland ecosystems [23], which potentially allow them to overcome the poor and spatial heterogeneous soil environments of these ecosystems. We determined if the plant root traits (i.e. root biomass, relative growth rate (RGR), AM colonisation, root acid phosphatase activity and N_2_ fixation) responded differently to soil nutrient heterogeneity or homogeneity using nitrogen supply. We also determined the responses of root traits to the inoculation of heterogeneous or homogeneous soils with AM fungi.

## Results

### Effects of AM fungi inoculation and nutrient distribution on root traits

**Fig. 1:**
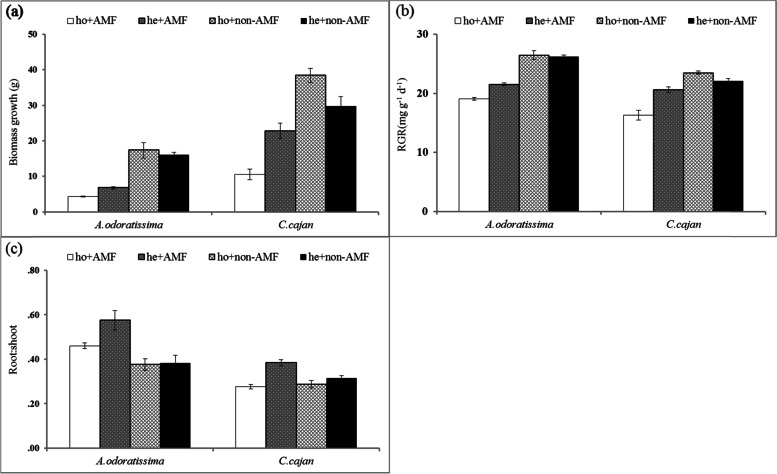
Biomass **(a)**, relative growth rate (RGR) **(b)** and root:shoot **(c)** response of two N_2_-fixing plant species (*Albizia odoratissima* and *Cajanus cajan*) grown in different soil conditions (ho, homogeneous; he, heterogenous; AMF, inoculated with arbuscular mycorrhizal fungi; non-AMF, not inoculated)

The effects of AM fungi and nutrient distribution on the whole biomass, RGR and root:shoot ratio were complex, and all interactions were significant (Table 1). First, the biomass, RGR and root:shoot values of *A. odoratissima* and *C. cajan* grow in AM fungi inoculated soil were higher in the heterogeneous soil environment than in the homogeneous soil environment (Fig. 1). Second, the biomass and RGR of both species were higher in uninoculated soil than in inoculated soil (Fig. 1b,c), regardless of the soil environment being heterogeneous or homogeneous; however, the opposite trend was observed in the root: shoot ratio (Fig. 1c).

**Table 1:** Effect of plant species, nutrients distribution and AM fungi addition on the RPA, RGR, biomass, and root: shoot ratios

Response variable	Linear mixed models	AIC	BIC	*T* value	Significant Difference
RPA	Null model	90.00	95.07	19.15	a
	Plant species	90.63	97.39	14.40	a
	Nutrients distribution	90.31	97.07	14.77	a
	AMF model	86.08	92.84	16.33	**b**
	Plant species * Nutrients distribution	90.58	99.03	12.88	a
	Species * AMF	84.88	93.32	15.78	**c**
	Nutrients distribution * AMF	84.01	92.46	16.75	**c**
	Plant species * Nutrients distribution * AMF	80.55	90.68	20.44	**d**
RGR	Null model	157.01	162.08	18.00	a
	Plant species	157.45	164.20	13.80	a
	Nutrients distribution	158.68	165.44	11.67	a
	AMF model	150.79	157.55	22.02	**b**
	Plant species * Nutrients distribution	159.05	167.49	10.25	a
	Plant species * AMF	147.32	155.76	24.38	**c**
	Nutrients distribution * AMF	151.85	160.29	16.95	**b**
	Plant species * Nutrients distribution * AMF	147.32	157.46	20.86	**c**
Biomass	Null model	256.21	261.27	4.44	a
	Plant species	253.74	260.50	2.34	**b**
	Nutrients distribution	258.18	264.94	2.81	a
	AMF model	253.73	260.48	5.34	**b**
	Species * Nutrients distribution	255.70	264.14	1.66	a
	Plant species * AMF	244.66	253.10	5.72	**c**
	Nutrients distribution * AMF	255.69	264.13	3.89	a
	Plant species * Nutrients distribution * AMF	246.49	256.62	4.33	**c**
Root:shoot	Null model	-91.82	-86.75	10.95	a
	Plant species	-95.69	-88.94	12.15	**b**
	Nutrients distribution	-90.84	-84.08	7.00	a
	AMF model	-91.67	-84.91	7.16	a
	Plant species * Nutrients distribution	-95.99	-87.55	9.71	**b**
	Plant species * AMF	-98.20	-89.76	10.88	**c**
	Nutrients distribution * AMF	-90.97	-82.53	5.25	a
	Plant species * Nutrients distribution * AMF	-100.81	-90.68	10.36	**d**

(1) Null model: ƒi = α + bi × Zgroups + εi; (2) Nutrients distribution or Plant species model: ƒi = α + β × X (Nutrients distribution or Plant species) + bi × Zgroups + εi, (3) Nutrients distribution * Plant species model: ƒi = α + β1 × X Specie + β2 × X Nutrients distribution + bi × Zgroups + εi. Here, ƒi represents response variable, α represents model intercept, bi represents random factor parameter, Z represents random effect, β represents fixed factor parameter, X represents fixed effect, and εi represents the unexplained effect. The minimum *AIC* (Akaike Information Criterion) and *BIC* (Bayesian Information Criterion) was defined as the optimal model. The null model was mainly used to estimate the effect of random factor on random factor, and other models were used to the effect of random and mixed factors. If letters in the last column were different, the mixed factors (Plant species or Nutrients distribution) were captured into these models

### Accumulation strategy of AM colonisation and root biomass in plant nutrient acquisition in heterogeneous soil

**Fig. 2:**
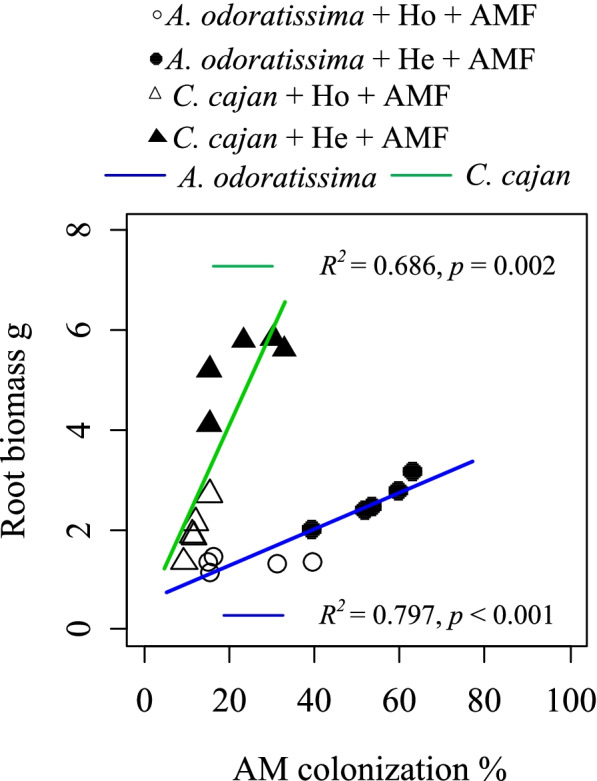
Relationship between root biomass and arbuscular mycorrhizal fungi (AMF) colonisation of two N_2_-fixing plant species (*Albizia odoratissima* and *Cajanus cajan*) grown in different soil conditions (ho, homogeneous; he, heterogenous; AMF, inoculated; non-AMF, not inoculated)

**Fig. 3:**
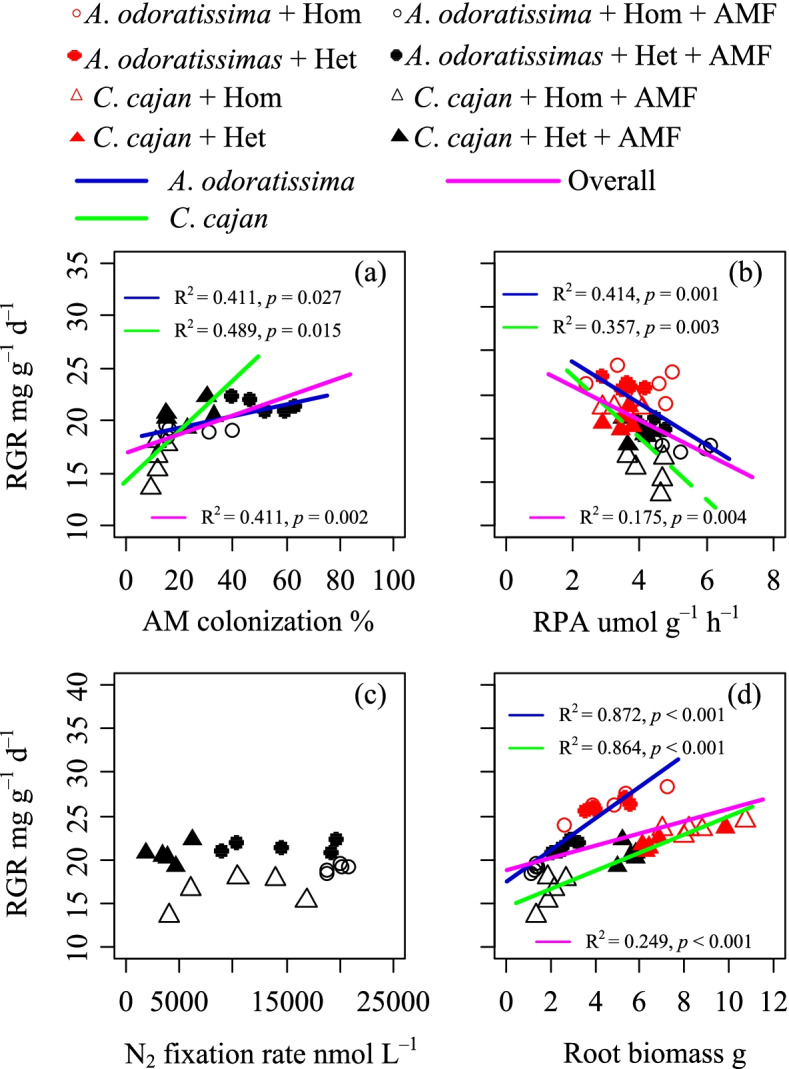
Relationship between the arbuscular mycorrhizal fungal colonisation and relative growth rate **(a)**; relative growth rate and root phosphatase activity phosphatases **(b)**; N_2_ fixation and relative growth rate **(c)**; root biomass and relative growth rate **(d)** of two N_2_-fixing plant species (*Albizia odoratissima* and *Cajanus cajan*) grown in different soil conditions (ho, homogeneous; he, heterogenous; AMF, inoculated; non-AMF, not inoculated)

AM fungi and roots are important for plant nutrient acquisition. A positive correlation between AM colonisation and root biomass was found in both the N_2_-fixing plants (*A. odoratissima: p* < 0.002, *R*^*2*^ = 0.686; *C. cajan*: *p* < 0.001, *R*^*2*^ = 0.797; Fig. 2). The RGR was positively correlated with the AM colonisation of both N_2_-fixing plants (*A. odoratissima: p* < 0.05, *R*^*2*^ = 0.411; *C. cajan*: *p* < 0.05, *R*^*2*^ = 0.489; Fig. 3a), and the root biomass of both N_2_-fixing plants (*A. odoratissima: p* < 0.001, *R*^*2*^ = 0.872; *C. cajan*: *p* < 0.001, *R*^*2*^ = 0.864; Fig. 3d). However, the RGR was negatively correlated with the root phosphatase activity of both N_2_-fixing plants (*A. odoratissima: p* < 0.05, *R*^*2*^ = 0.414; *A. odoratissima: p* < 0.05, *R*^*2*^ = 0.357; Fig. 3b). The N_2_ fixation rate was not correlated with the RGR.

### Correlations of AM colonisation, N_2_ fixation rate and phosphatases in heterogeneous and homogeneous soil environments

**Fig. 4:**
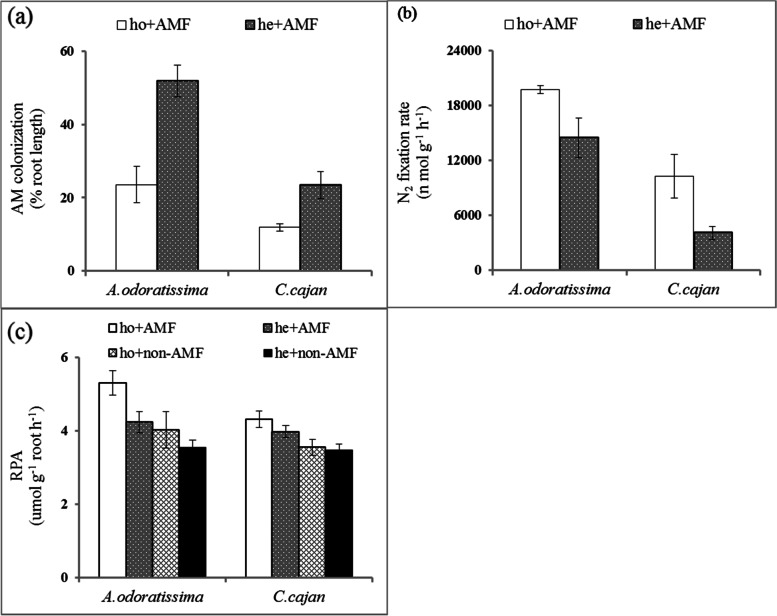
Arbuscular mycorrhizal fungi (AMF) colonisation **(a)**, N_2_ fixation **(b)** and root phosphatase activity (RPA; **c)** response of two N_2_-fixing plant species (*Albizia odoratissima* and *Cajanus cajan*) grown in different soil conditions (ho, homogeneous; he, heterogenous; AMF, inoculated; non-AMF, not inoculated)

**Fig. 5:**
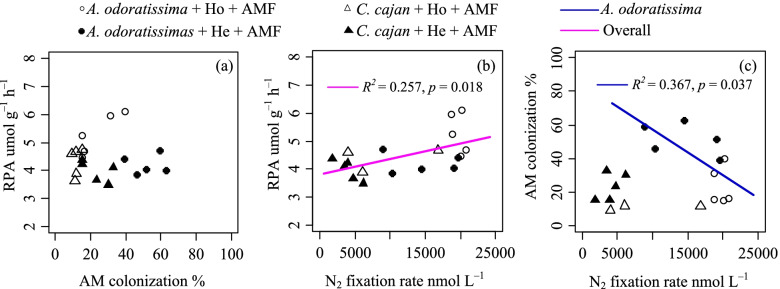
Relationship among arbuscular mycorrhizal fungal colonisation and root phosphatase activities **(a)**; N_2_ fixation rates and root phosphatase activities **(b)**; arbuscular mycorrhizal fungal colonisation and N_2_ fixation rate **(c)** of two N_2_-fixing plant species (*Albizia odoratissima* and *Cajanus cajan*) grown in different soil conditions (ho, homogeneous; he, heterogenous; AMF, inoculated; non-AMF, not inoculat

Given the effect of soil nutrient distribution on plant growth, we determined if the plants used AM fungi, fixation and phosphatases to overcome the nutrient acquisition issue associated with a heterogeneous soil environment. Root phosphatase activity, AM colonisation and N_2_ fixation varied among the heterogeneous and homogeneous soil environments (Table 2). When grown in AM fungi inoculated soil, *A. odoratissima* had a higher N_2_ fixation rate and AM colonisation than that of *C. cajan*, regardless if the soil environment was heterogeneous or homogeneous (Fig. 4a, b). AM colonisation of *A. odoratissima* and *C. cajan* was higher (Fig. 4a) and the N_2_ fixation rates were lower (Fig. 4b) in the heterogeneous soil environments than in the homogeneous soil environments. Both *A. odoratissima* and *C. cajan* grown in AM fungi inoculated soil produced more phosphatase and maintained higher root phosphatase activity in the homogeneous soil environment comparing to the heterogeneous soil environment (Fig. 4c). Root phosphatase activities of *A. odoratissima* and *C. cajan* were higher when grown in AM fungi inoculated soil than in uninoculated soil in both heterogeneous and homogeneous soil environments (Fig. 4c). The root phosphatase activity was positively correlated with the overall N_2_ fixation rate (*p* < 0.01, *R*^*2*^ = 0.257; Fig. 5b), but not significantly correlated with the AM colonisation (Fig. 5a). AM colonisation was negatively correlated with the N_2_ fixation rate of *A. odoratissima* (*p* < 0.05, *R*^*2*^ = 0.367; Fig. 5c).

**Table 2:** Effect of nutrients distribution on the N_2_ fixation rate and AM colonisation

Response variable	Linear mixed models	AIC	BIC	*T* value	Significant Difference
N_2_ fixation rate	Null model	401.65	404.64	3.66	a
	Plant species	398.14	402.12	5.93	**b**
	Nutrients distribution	402.50	406.48	3.02	a
	Plant species * Nutrients distribution	391.40	396.38	14.19	**c**
AM colonisation	Null model	159.24	162.23	3.26	a
	Plant species	158.75	162.73	3.50	a
	Nutrients distribution	158.81	162.79	1.64	a
	Plant species * Nutrients distribution	153.16	158.14	3.84	**b**

(1) Null model: ƒi = α + bi × Zgroups + εi; (2) Nutrients distribution or Plant species model: ƒi = α + β × X (Nutrients distribution or Plant species) + bi × Zgroups + εi, (3) Nutrients distribution * Plant species model: ƒi = α + β1 × X Specie + β2 × X Nutrients distribution + bi × Zgroups + εi. Here, ƒi represents response variable, α represents model intercept, bi represents random factor parameter, Z represents random effect, β represents fixed factor parameter, X represents fixed effect, and εi represents the unexplained effect. The minimum *AIC* (Akaike Information Criterion) and *BIC* (Bayesian Information Criterion) was defined as the optimal model. The null model was mainly used to estimate the effect of random factor on random factor, and other models were used to the effect of random and mixed factors. If letters in the last column were different, the mixed factors (Plant species or Nutrients distribution) were captured into these models

## Discussion

### Accumulation strategy of AM fungi and roots in plant nutrient acquisition

Increasing evidence supports the idea that root traits (e.g. root biomass) exhibit wide variations in heterogeneous and homogeneous soil environments [12, 13], which strongly influence the colonisation of AM with roots [10, 24, 25]. Our results showed that the plants had a higher root biomass and AM colonisation in the heterogeneous soil environment than in the homogeneous soil environment (Figs. 1c and 4a). AM colonisation was positively correlated with root biomass in the heterogeneous soil environment (Fig. 2). These results indicated that accumulation strategies involved in belowground resource acquisition existed between the roots and their associated AM fungi, which benefitted the nutrient acquisition of plants for growth in the poor and heterogeneous of soil environment of karst shrubland ecosystems.

The accumulation strategies of plant nutrient acquisition between root biomass and AM fungi in poor and heterogeneous soil environments can be explained by the root absorption capacity [26]. For example, during seedling establishment in soil with low and heterogeneous levels of P, as in our study (soil available P content 1.92 mg kg^−1^), plant species increase their root absorption area as a strategy to increase their acquisition of soil nutrients. Larger absorption areas of the roots can be induced in more heterogeneous soil environments. Many previous studies have indicated that plants grown in heterogeneous soil environments preferentially partition more photosynthetic products to underground parts [12, 13], and further increase their root biomass. More roots increase the absorption area of the roots, which benefits plant nutrient capture [6]. Furthermore, a large absorption area of the roots allows the roots to amplify the interface contact with soil hyphae, and thus increase the chance of attracting symbiotic AM fungi. Simultaneously, more roots can exude a sizeable quantity of polysaccharides [27], which can invest more C for AM colonisation and indirectly promote nutrient acquisition. Thus, a large absorption area of the roots can be gained by AM colonisation. Higher levels of AM colonisation can be found in heterogeneous soil environments compared with homogeneous soil environments [14], as found in the present study, which potentially enhances the nutrient acquisition for plant root growth. Importantly, the hyphae of AM can extend between rocks to reach areas that are not accessible to plant roots [28]. This characteristic is a very efficient way to generate absorptive surfaces for plant growth in karst regions with a high rock-soil ratio. Thus, increasing the AM colonisation and root biomass are two important strategies to construct absorptive surface areas for plants to adapt to nutrient poor and heterogeneous soils. Therefore, our results were consistent with the hypothesis that higher levels of AM colonisation and a larger root biomass increases the absorptive surface for plant nutrient acquisition, to maintain a high growth rate of plants in nutrient poor and heterogeneous soil of karst shrubland ecosystems.

AM fungi symbiosis with root enables plants better acquirement soil nutrients, and greatly affect their plant growth. Many previous studies reported that plant growth responds different to inoculation AM fungi [29, 30]. Our results showed that plant growth responses were negatively correlated with the inoculation of soil with AM fungi, independent of the heterogeneity or homogeneity of the soil environment. These findings were consistent with those of Zhang et al. [31], but inconsistent with those of Liang et al. [29] who demonstrated positive effects on plant growth when inoculated AM fungi. Several possible reasons could explain this phenomenon.

First, differences in the plant functional group could influence the AM colonisation. For example, plants have coarse root architecture, including short root hairs, large root diameters and low root hair densities, are positively correlated with the plant growth responses to inoculation with mycorrhiza [4, 10]. Certain plants have limited intrinsic abilities to acquire nutrients [31], and they thus obtain nutrients mainly depending on AM fungi. In contrast, AM fungi colonisation is only an alternative for plant species with fine root architecture (e.g. greater root density and hair length) to absorb nutrients [10]. Thus, plant growth of this kind of plant would be negatively correlated with the inoculation of mycorrhiza. The present study only assessed the growth responses of N_2_-fixing plants to AM fungi inoculation of the soil environment. Thus, the growth responses of plant functional groups, such as N_2_-fixing plants and non-N_2_-fixing plants, to AM fungi inoculation of the soil environment should be assessed and compared in future studies.

Second, the plant growth responses to AM fungi inoculation were related to the species-specific interactions between AM fungi and the host plant. Some previous studies focused on inoculation AM fungi influencing plant growth mainly through the inoculation of commercial AM fungi strains [32, 33]. Commercial AM fungi strains that are not optimally matched to the host plant lead to lower AM colonisation rates and less benefit from AM fungi. This action reduces the diversity of AM fungal species, which may play key roles in plant growth and even have negative effects on plant growth. The AM fungi colonisation of plants growing in natural soil was determined in the present study, which served as the best representative of the soil biota pool, including the total AM fungi. Therefore, plants were exposed to their natural AM fungi assemblages, which may have increased the actual benefits of the plant-AM fungi symbiosis. However, a negative effect of mycorrhizal inoculation on plant growth was found in the present study, which was related to the soil pathogens. Plants not only show host-specificity in symbiotic relationships with beneficial microbes (i.e. AM fungi) [34, 35] but also share the same associations with pathogens [36]. Pathogens have negative effects on plant growth by inducing higher disease mortality rates in the plants [29, 36], or competing with the plants for carbohydrates [37, 38]. The treatment soil (e.g. without AM inoculate) was sterilised in the present study, which killed all pathogens and thus would enable the promotion of plant growth independent of soil heterogeneity and homogeneity (Fig. 1). The same findings were found by Zhang et al. [30]. Although plant growth was not significantly improved by the AM inoculation in the present study, it increased the root biomass in the heterogeneous soil environment compared with homogeneous soil environment. These findings suggested that plants would strengthen their symbiosis with AM, and then allocate more C to the roots and for hyphal production in heterogeneous soil environments, with a higher investment in root growth at the expense of shoot growth [39–41]. Similar results have been reported by previous studies, whereby plants increase their root:shoot ratio to enhance nutrient absorption in heterogeneous soil environments [42, 43].

### Roles of AM fungi in N and P nutrient acquisition in karst shrubland ecosystems

AM fungi are well known to improve plant phosphorus nutrients, especially under low phosphorus conditions. For example, AM fungi improve the absorption of soil inorganic phosphorus through hyphae. Simultaneously, fungi produce phosphatase enzymes [5, 44], which mineralise more organic phosphorus from ester-bound forms to the orthophosphate form to increase the plant uptake [19–21]. Therefore, AM fungi and root phosphatase enzymes are two vital phosphorus acquisition strategies for plants [5, 45]. The results of the present study showed that AM colonisation and root phosphatase enzyme activity did not significantly correlation in heterogeneous and homogeneous soil environments (Fig. 3a). These results suggested that AM fungi most likely enhanced inorganic phosphorus acquirement in the heterogeneous soil environment of karst shrubland ecosystems, which is consistent with as the previous studies reported [46, 47].

The AM colonisation-N_2_ fixation tripartite symbionts also played important roles in plant nutrient acquisition. The AM colonisation-N_2_ fixation tripartite symbionts were much more beneficial for plant growth in natural nutrient limitation environments (e.g. N and P) [48–50]. A negative correlation between AM colonisation and N_2_ fixation of *A. odoratissima* was found in the heterogeneous soil environment (Fig. 5c). This relationship can be explained by the complementary strategies between AM fungi and N_2_-fixing symbionts in the nutrient acquisition of nitrogen and phosphorus. Nutrient acquisition strategies, including those of AM fungi and rhizobia, cost a large amount of resource [45, 51]. From a cost–benefit perspective, plants select strategies that maximise the benefits while minimizing the costs [52]. Therefore, when nitrogen was abundantly available in the present study, plants absorbed nitrogen directly through their roots with less resource, and further reduced the N_2_ fixation. Thus, plants mainly depend on AM for the acquisition of phosphorus in low phosphorus soils, and more C is invested for AM colonisation. However, no correlation between AM colonisation and N_2_ fixation was detected in *C. cajan.* Therefore, the relationship between AM colonisation and N_2_ fixation is more complex, and it is still unclear if fixing N_2_ is necessary to acquire phosphorus or vice versa.

## Conclusions

In this study, accumulation strategies between roots and AM fungi were shown to exist in the belowground nutrient resource foraging of N_2_-fixing plants in a karst shrubland ecosystem. The N_2_-fixing plants grown in nutrient poor and heterogeneous soil environments relied more on AM fungi and an increased absorptive root surface for the acquisition of nutrients. This result suggested that an increase in the AM colonisation of roots and an increase in root biomass beneficially increased the absorptive surfaces for the acquisition of nutrients under conditions of spatially heterogeneous soil nutrient availability. Plants grown in soil inoculated with AM could increase their root-shoot ratio to a higher degree in the heterogeneous soil environment than that in the homogeneous soil environment. AM fungi and N_2_ fixing symbionts play important roles in plant nutrient acquirement. However, the relationship between AM colonisation and the N_2_ fixation rate differ in the two N_2_-fixing plants, which indicated that host-specificity characteristics influence the nutrient acquisition strategies of plants. Our findings suggested that plants regulate root-mycorrhizal interactions to adapt to the nutrient poor and heterogeneous soil environments of karst shrubland ecosystems. Future studies combining plant functional groups with AM fungal species to measure how soil conditions, mycorrhizal type and root traits (e.g. root length and density) can collectively mediate resource acquisition strategies in belowground.

## Methods

### Experimental design

We conducted a potted experiment in Huanjiang Observation and Research Station for Karst Ecosystems of Institute of Subtropical Agriculture, the Chinese Academy of Sciences, Southwest of China (24°44' N, 107°51' E). Soils for all treatments were collected from a shrub ecosystem located in the Huanjiang Observation and Research Station, and were classified as calcareous lithosols (limestone soil) basis for the FAO/UNESCO classification system [53]. Soils were sieved through a 5 mm mesh and stored at 4 °C in the dark before experimentation. Karst soil has relatively low contents of total nitrogen (4.48 g kg^−1^) and availability phosphorus (1.92 mg kg^−1^) compared with that of other non-karst regions [36, 38].

The experiments were conducted using a random factorial design including three factors. (1) Plant species: two common N_2_-fixing shrub species were used, *A. odoratissima* and *C. cajan*, which are described in detail in Table 3. *A. odoratissima* and *C. cajan* in the present study are identified by Fujing Pan professor according to Flora Reipublicae Popularis Sinioae (http://frps.iplant.cn). Herbarium of *A. odoratissima* was deposited by Liang Xiangri (voucher number: IBK00067946; https://www.cvh.ac.cn/spms/detail.php?id=c0a9455f), and Herbarium of *C. cajan* was deposited by Xu Yuebang (voucher number: IBK00069967; https://www.cvh.ac.cn/spms/detail.php?id=c0ae354c). Plant samples have been permitted to collect [see Additional files 1]. Both of these species are the most widespread and abundant species of symbiotic fixers across karst shrub ecosystems [36]. (2) Spatial distribution of nutrients: two soil nutrient spatial distributions (homogeneous and heterogeneous) were created via the addition of nitrogen. (3) two levels of AM fungi, AM inoculation or not. Plants were grown in either all of sterilised soils (without AM fungi inoculation) or all of non-sterilised soils (inoculated with AM fungi). Soils were sterilised in an autoclave oven for 1 h at 120 °C [55]. To ensure the effectiveness of the sterilization procedure and plants without-inoculated AM, AM colonisation was determined in the sterilized soils after plant harvest. A total of 56 samples were used, comprising 7 replicates of each of the 8 treatments. Finally, 5 replicates with similar plant growth potentials were harvested, resulting in a total of 40 analysed samples.

**Table 3:** The two shrub species used in the experimental studies organized by family, functional group, initial mass and geographic distribution (data came from Flora Reipublicae Popularis Sinioae, http://frps.iplant.cn)

Species	Family	Functional group	Distribution	Initial mass (g)
*Albizia odoratissima* Benth	Albizia	N_2_ fixer	Southwest China	0.15
*Cajanus cajan* Mill sp	Cajanus	N_2_ fixer	Southwest and southeast China	0.56

**Fig. 6:**
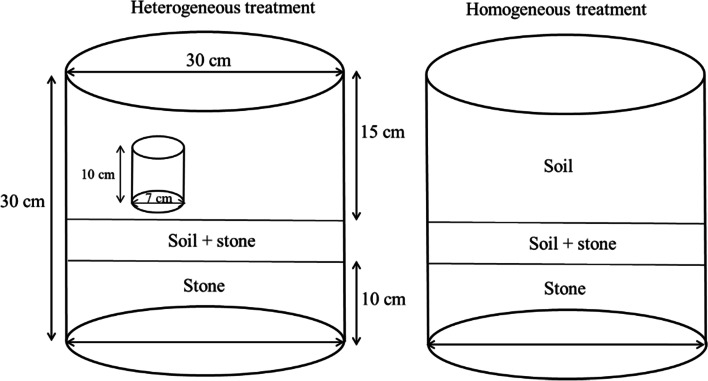
Schematic representation (not drawn to scale) of the pots used in the experiment according to García-Palacios et al. [6] revision. In the heterogeneous treatments, a plastic cylinder was filled with the nitrogen nutrient

Seeds of *A. odoratissima* and *C. cajan* were collected from the nearby shrubland. The seeds were sterilised with 10% household bleach solution (1 min), washed with distilled water and then sown in plastic cups (200 ml) at homogenised sterilised soil. Three weeks after sowing, two seedlings with similar growth potentials were transplanted into one pot (depth 30 cm, diameter 30 cm). After transplantation for 1 week, dead or poorly growing seedlings (due to injuries during the transfer) were removed. To simulate the soil layers in karst regions, plots included three layers (Fig. 6). Plastic cylinders consisting of a light mesh were placed within the pots in the heterogeneous plot, while no plastic cylinders were placed in the pots in the homogeneous plot (Fig. 6). Nitrogen was supplied as NH_4_Cl (10 g N m^−2^ year^−1^), because it is reportedly the limiting factor for shrub growth in karst shrub ecosystems [23]. Nutrients were injected into the plastic cylinders through plastic tubes in the heterogeneous plot, and were evenly added to the surface soil in the homogeneous plot. Plants were watered gravimetrically by weighing each plot every 2 d. Overall, 15 seedlings per species were harvested to measure the initial biomass [ranging from 0.13 g (*A. odoratissima*) to 0.51 g (*C. cajan*); Table 3].

After 15 weeks (September 2018), all study plants were harvested and divided into leaves, stems and roots. Roots were washed with deionised water. Some root samples were divided into two parts: one part was stored in 95% ethanol for AM colonisation analysis; the other part was stored at -20 °C for root phosphatase activity analysis. The remaining roots, leaves and stems per plants were dried at 65 °C for 60 h to measure the plant biomass, root-shoot allocation and the content ratio of tissue carbon: nitrogen: phosphorus. The relative growth rate (RGR) was calculated as follows:

$$\mathrm{RGR}=\left({\mathrm{log}}_{\mathrm{e}}\left({\mathrm{M}}_{\mathrm{f}}\right)-{\mathrm{log}}_{\mathrm{e}}\left({\mathrm{M}}_{\mathrm{i}}\right)\right)/{\mathrm{d}}_{\mathrm{t}}.$$

where, M_i_ is the initial mass, M_f_ is the final mass and d_t_ is the duration days of experiment [5].

Nitrogen fixation rates were determined using an acetylene reduction assay [55]. During the harvesting of the N_2_-fixing plants, fresh nodules were excised from N_2_-fixing plant roots and incubated with a conical flask (125 ml; with 10% acetylene atmosphere) for 30 min in situ. A 30 ml gas samples were extracted from the sealed conical flasks using syringe vials after incubation, injected to 12 ml pre-evacuated glass vials (LabcoExetainer, Labco Limited, UK). The samples were transferred to laboratory and analyzed by gas chromatograph (Agilent GC 7890A, Agilent, USA) equipped with a flame ionization detector.

Acid phosphatase (phosphomonoesterase) activity in the root samples was measured following Sinsabaugh et al. [56]. Sodium bicarbonate (50 mM, pH 5.0) was used as the buffer. Briefly, the 12 wells were assigned to the sample assay (0.8 ml buffer + 20 ~ 30 g roots + 200 μl 200 μM MUB-linked substrate), soil control (1 ml buffer + 20 ~ 30 g roots), negative control (0.8 ml buffer + 200 μl 200 μM MUB-linked substrate), reference standard (0.8 ml buffer + 200 μl 10 μM 4-MUB solution), quench standard (0.8 ml buffer + 20–30 g roots + 200 μl 10 μM 4-MUB solution) and blank wells (1 ml buffer). These 12-well plates were shaken (110 rpm) for 1 h at 25 °C. Each well was added 10 ml NaOH (1.0 M) to stop the reaction. Subsamples (200 μl) from each replicate were pipetted into a black 96-well microplate and were measured by microplate fluorometer (Infinite 200 Pro, Tecan, Switzerland) at 450 nm emission and 365 nm excitation. The μmol 4–4-MUB-P g^−1^ root h^−1^ was used to calculate root phosphatase activity.

AM colonisation was measured following Phillips and Hayman [57]. Fine roots (1 cm diameter) of each root sample were cleaned with KOH (10%; w/v), and stained with trypan blue (0.05%; v/v) for the quantification of root colonisation according to the magnified intersection method by McGonigle et al. [58].

### Statistical analysis

Before statistical analysis, data (i.e., AM colonisation, biomass, RGR, root phosphatase activity and N_2_ fixation) were log-transformed to conform to normality (IBM Corp., Armonk, NY, USA). The *p*-values < 0.05 were considered as Statistical significance. A two-sample t-test was used to analyse the differences in AM colonisation, biomass, RGR, root: shoot ratios, root phosphatase activity and N_2_ fixation between heterogeneity and homogeneous soil environments with inoculated or uninoculated soil (Figs. 1 and 4). Pearson’s correlations were used to test the relationships among AM colonisation, biomass, RGR, root phosphatase activity and N_2_ fixation (Figs. 2, 3 and 5). The mixed effect models (lme4 package with R) were used to analyse the effects of plant species, soil condition and AM fungi inoculation on the variance in biomass, RGR, root phosphatase activity and root: shoot ratios [59]. The soil condition, AM fungi inoculation and plant species were modelled as fixed factors, and all repeated samples could be divided into eight groups and as random factors (Tables 1 and 2). We also assessed the relationships between AM colonisation, biomass, N_2_ fixation and phosphatases, and presented them as scatter plots (Figs. 2 and 5).

## Supplementary Information

**Additional file 1.**

## Data Availability

The datasets generated during and/or analyzed during the current study are available from the corresponding author on reasonable request.

## Abbreviations

AMF: Arbuscular mycorrhizal fungi
RGR: Relative growth rate
A. odoratissima: Albizia odoratissima
C. cajan: Cajanus cajan
