# Supplementary figures and images for: Accumulation in nutrient acquisition strategies of arbuscular mycorrhizal fungi and plant roots in poor and heterogeneous soils of karst shrub ecosystems
Source: BMC Plant Biol. 2022 Apr 11;22:188. doi: 10.1186/s12870-022-03514-y (PMC8996662; doi:10.1186/s12870-022-03514-y)

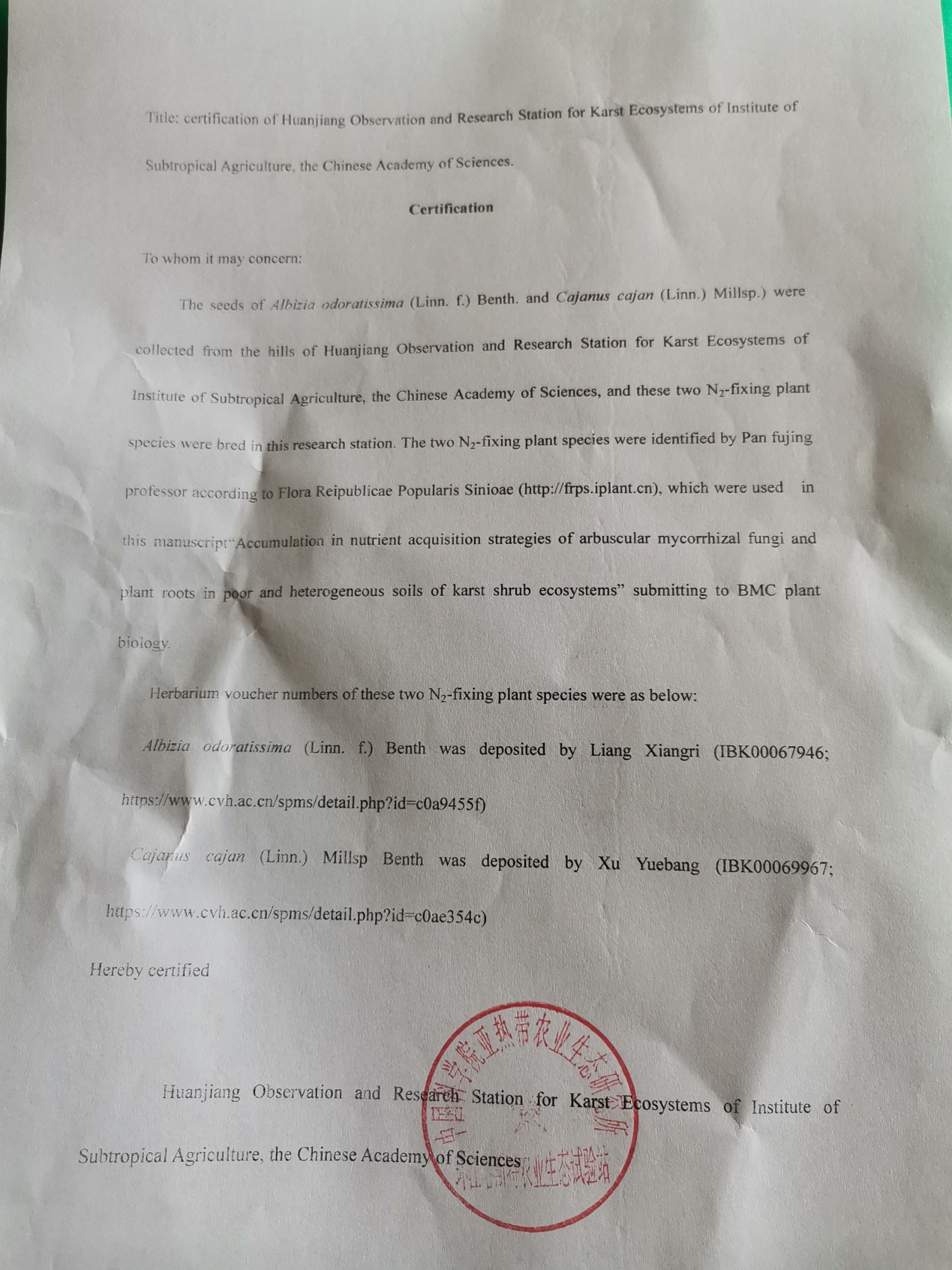

Supplement: Supplementary file 1 — Additional file 1. [file 12870_2022_3514_MOESM1_ESM.docx]
